# Remedies and Their Uses

**Published:** 1906-11-03

**Authors:** 


### REMEDIES AND THEIR USES.

### Valyl Hœchst.

THERE are perhaps no more difficult cases for the general practitioner than those grouped under the headings of hysteria, neurasthenia, and functional disturbance. In many of the functional conditions common in women, especially at the climacteric, valerian has been found a valuable drug, and it has certainly stood the test of time. As, however, is the case with nearly all such preparations, its composition, and consequently its pharmacological action, is variable; and investigations have consequently been undertaken with a view to determining its active constituents and administering them in a pure state. The essential oil contains certain terpenes and acids of which Kionka and Liebrecht consider valerianic acid the most important from the point of view of physiological action. Its use as a drug is, however, rendered impossible by its toxic properties, whilst its salts as a rule are more or less inert. After investigating the properties of a number of its organic derivatives, they found that valeryl-diethyl-amide, $CH_3(CH_2)_3CON(C_2H_5)_2$, was the most valuable medicinally and produced the characteristic effects of valerian. Messrs. Meister Lucius and Brüning have introduced this substance under the name of valyl Hœchst. Owing to its burning taste and its tendency to oxidation it is dispensed in gelatine capcules containing two grains each. As a rule two or three of these capsules should be given thrice daily. No by-effects have been observed in most cases, even when eight or ten capsules were taken at once, or six thrice daily. Eructations of a "fruity" character have occasionally been observed, and exceptionally heartburn has occurred. The action of the drug on the central nervous system and the psychic centres appears to be very similar to that of valerian root; it has a definite effect in raising the peripheral blood-pressure owing to vaso-constrictor stimulation, with subsequent distension of the peripheral vessels, hyperæmia of the internal vessels, and fall of general blood-pressure.

The main indications for the administration of valyl Hœchst are the same as those for the old tincture of valerian. Cases have been recorded in which it has done good in hysteria, neuralgia, neurasthenia, and the traumatic neuroses, especially when attributable to vasomotor disturbances. Klemperer (*Therap. du Gegenwart,* January 1902) published a series of cases in which he noted good results. In twelve cases of "nervous excitement" valyl in the majority seemed to act better than infusion of valerian root. In purely nervous cardiac disturbances he found the results excellent, but true angina pectoris was unaffected. In some cases of migraine and nervous insomnia it did good, but here the effect was not so invariable; and in "nervous dyspepsia" the pain appeared to be aggravated. Freudenberg (*Frauenarzt.,* May 1902) has published a series of cases mainly of "nervous disturbance," in all of which he observed good effects. Insomnia, migraine, and nervous gastralgia were among the cases successfully treated by him. Numerous other papers on the subject have appeared since its introduction, and the general opinion seems to be that valyl Hœchst is a valuable drug.

